# Group-specific effect of interannual water level fluctuation on consumers trophic niche area

**DOI:** 10.1038/s41598-026-37620-w

**Published:** 2026-02-05

**Authors:** Lukáš Veselý, Timo J. Ruokonen, Fabio Ercoli, Martin Bláha, Miloš Buřič, Paride Balzani

**Affiliations:** 1 Faculty of Fisheries and Protection of Waters, University of South Bohemia in České Budějovice, South Bohemian Research Center of Aquaculture and Biodiversity of Hydrocenoses, Zátiší 728/II, Vodňany, 38925 Czech Republic; 2 Natural Resources Institute Finland, Survontie 9 A, 40500 Jyväskylä, Finland; 3 Institute of Agricultural and Environmental Sciences, Chair of Hydrobiology and Fisheries, Estonian University of Life Sciences, Kreutzwaldi 5D, 51006 Tartu, Estonia; 4 Department of Biological and Environmental Science, University of Jyväskylä, P.O. Box 35, FI-40014 Jyväskylä, Finland

**Keywords:** Stable isotopes, Carbon, Nitrogen, Fish, Crayfish, Macrozoobenthos, Ecology, Freshwater ecology, Stable isotope analysis

## Abstract

The trophic niche area defines the consumer’s ability to utilize a range of food sources and their role in community. However, the trophic niche area might be influenced by a variety of biotic and abiotic factors. For instance, water level fluctuation might also affect the trophic niche area of all consumers inhabiting a given aquatic ecosystem. Thus, in this study we investigated the effect of water level fluctuation on the isotopic niche area of multiple consumers (macroinvertebrates and fish) belonging to different trophic guilds (i.e., predator, omnivore, and detritivore) over three years. The results showed that water level fluctuation strongly affects the isotopic niche area of all consumers. Nevertheless, the magnitude and direction were group specific. While predatory fish increased the isotopic niche area when the water level dropped, omnivorous fish experienced a decrease; these two trends continued even after the water level drop. In crayfish, the isotopic niche area was considerably larger during the year with water level drop. However, decreasing water levels resulted in a corresponding decrease in isotopic niche area in detritivores and predatory macrozoobenthos. Our study demonstrated the importance of water fluctuations on the isotopic niche area across different types of consumers.

## Introduction

The trophic niche area is an important functional trait defining the consumer’s ability to use a range of food sources and its role in the community^1^. In general, the trophic niche area varies among specialists (predators, herbivores) and generalists (omnivorous species), with the latter usually having a wider trophic niche area than the former^2,3^. Furthermore, the trophic niche area can vary spatially and temporally^4–6^, influenced by several biotic and abiotic factors^1,7^. Although this concept is crucial in food web ecology, the processes driving changes in consumers’ trophic niche area remain understudied.

The trophic niche can be proxied by carbon and nitrogen stable isotope signatures (δ¹³C and δ¹⁵N, respectively), where the former indicates the range of primary resources consumed and the latter the trophic position of the consumer^8^. These signatures can be used in a bivariate space (the δ-space) to investigate, inter alia, the isotopic niche area of a consumer^9^, allowing, for example, the comparison of isotopic niches within and among species and communities^10–12^. For example, Hayden et al.^1^ and Veselý et al.^13^ reported positive relationships between the size of consumers and their isotopic niche area, highlighting an ontogenetic increase in generalist feeding. In addition, non-native species have been shown to affect other consumers’ isotopic niche area^7,14–16^.

Among abiotic variables, temperature was shown to affect fish isotopic niche area in a species-specific way due to species temperature optima^17^. Temperature directly influences metabolic and feeding rates, thereby shaping how consumers exploit available resources. At moderate warming, increased metabolism can expand foraging activity and broaden the isotopic niche, whereas exposure to temperatures approaching or exceeding species thermal limits may reduce performance and dietary diversity, leading to a narrower niche^1,18^. Productivity also plays a role in trophic niche dynamics. Higher primary production can increase prey abundance and resource heterogeneity, potentially broadening consumers’ isotopic niches^1^. However, in highly productive systems, abundant prey item may promote dietary specialization, resulting in a narrower isotopic niche, as suggested by Lesser et al.^19^. This dual effect implies that the relationship between productivity and isotopic niche area can be context-dependent, varying with resource distribution and consumer guild.

Pollution, including chemical contamination and microplastic accumulation, may further influence trophic niche size by reducing quality prey item or availability and altering energy pathways. For instance, Giani et al.^20^ demonstrated that elevated microplastic concentrations were associated with narrower isotopic niches in marine fish, likely due to impaired feeding efficiency and reduced resource diversity. Similar processes may occur in freshwater ecosystems exposed to anthropogenic stressors.

Water level fluctuation is an important factor for many community and population aspects, such as biodiversity^21^, community composition and structure^22,23^, as well as consumers’ diet^7,24,25^ and behaviour^26^, while also influencing the ecosystem’s nutrient content^27^ or food source availability and habitat accessibility^28^. Beyond these general effects, low-water periods can substantially alter predator–prey dynamics and trophic structure. When the water level drops, aquatic organisms become confined to smaller volumes of habitat, which increases their local density, encounter rates, and competition for resources^29,30^. At the same time, habitat alteration such as the loss of vegetated littoral zones and other structural refuges enhances the vulnerability of prey to predators^31,32^. Consequently, low-water conditions often intensify predator–prey interactions and can shift consumers’ trophic positions. However, the direction and magnitude of these effects depend on trophic guild. For instance, highly mobile predators such as piscivorous fish may broaden their trophic niche as prey diversity and accessibility increase under habitat compression^30,33^, while benthic detritivores or invertivores may experience a contraction of their trophic niche due to the loss of shallow habitats and reduced detrital inputs^34^. Conversely, herbivores and omnivores often expand their niche during high-water phases, when flooding enhances access to macrophytes, periphyton, and other basal resources^35–37^. These contrasting responses are largely explained by differences in mobility, foraging strategy, and the degree of dependence on littoral versus pelagic resources.

In addition to natural seasonal variation, human activities such as dam construction and reservoir operation can amplify or alter the frequency and amplitude of water level fluctuations^38–40^. Artificial water level peaking can disrupt natural hydrological cycles, degrade littoral habitats, and reshape food webs by changing basal resource availability and consumer interactions^29,34,41^.

Previous studies showed that water level fluctuation can affect trophic web structure as a result of increased prey–predator interactions during low-water periods^29,30,32^, resulting in positive, negative, or no effects on the isotopic niche area of fish species, depending on the trophic guild^34–36^. For instance, Que et al.^30^ reported that during periods of low water levels, predator–prey interactions intensified due to increased predator densities resulting from reduced habitat availability, thereby driving piscivorous fish toward higher trophic positions. In addition, Eloranta et al.^25^ reported a narrower population niche in Arctic charr (*Salvelinus alpinus*) in a regulated Arctic lake (high amplitude of water level fluctuation), due to lack of truly littoral specialist individuals in comparison to an unregulated lake. Furthermore, Luo et al.^42^ observed narrower trophic niche areas during low water level and broader ones during high water levels, likely reflecting reduced and increased food source availability, respectively. Despite its potential ecological significance, the influence of water level fluctuations on isotopic niche area remains understudied across other taxa.

In this study, we used carbon and nitrogen stable isotope data to analyze interannual changes in the isotopic niche area of multiple consumers (macroinvertebrates and fish) belonging to different trophic guilds (i.e., predator, omnivore, and detritivore) in a reservoir. We compared the consumers’ isotopic niche area over a three-year period, during which a planned dam reconstruction caused a water level drop in the second year followed by recovery in the third year. We hypothesized that: (1) the water level drop (in the second year) shrank the isotopic niche area of all consumers compared to the first year, but (2) the magnitude of such variation will be consumer-specific.

## Materials and methods

### Study site

**Fig. 1:**
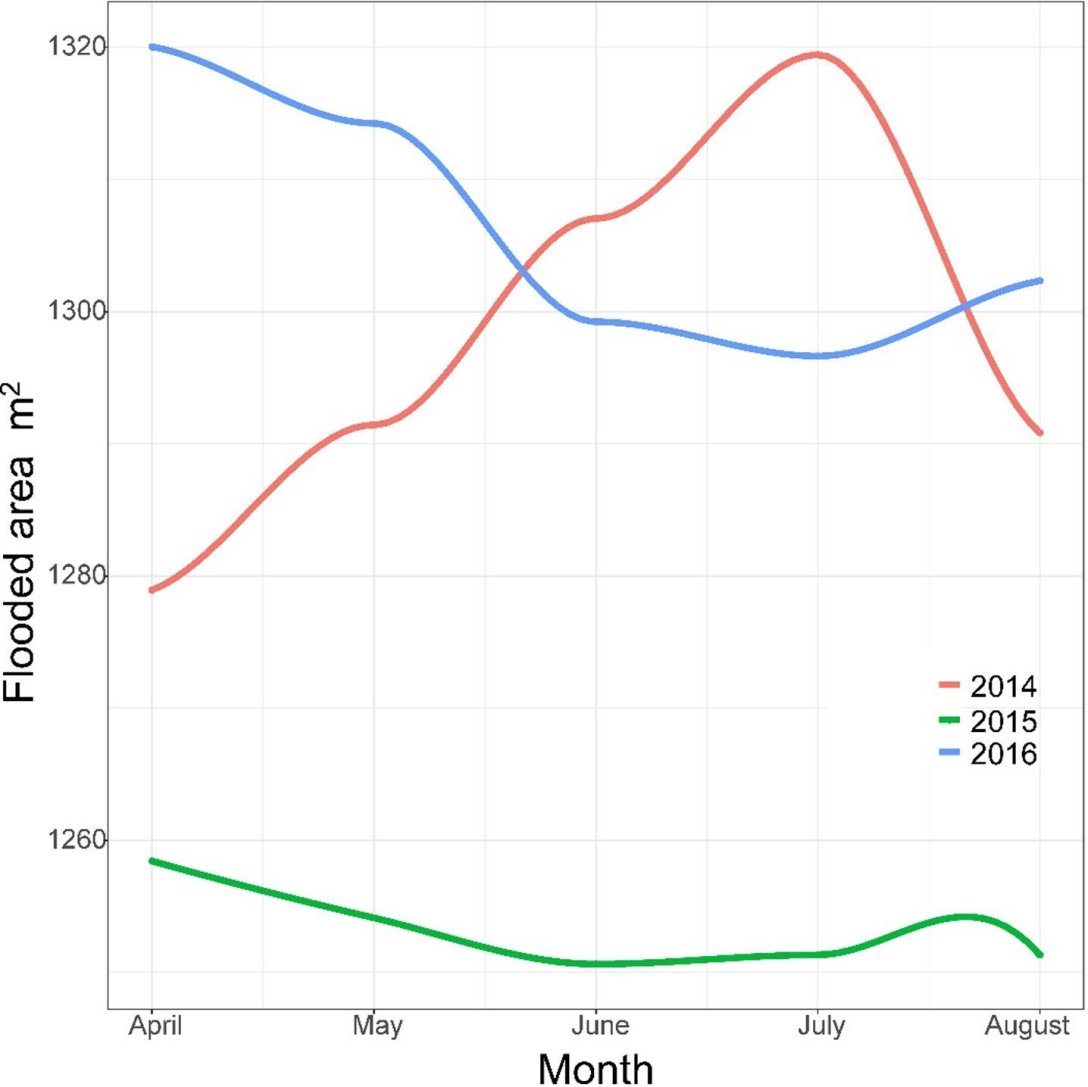
Flooded area of Nýrsko reservoir in years 2014–2016.

The Nýrsko reservoir is an oligotrophic water supply reservoir located in West Bohemia, Czechia (49°15′27″N, 13°8′46″E). The surface of the reservoir is 1.48 km² and its maximum depth is 34 m. The reservoir has a canyon-like shape, with the orographic left bank gradually sloping towards the bottom, which is composed of fine particles and covered by macrophytes, while the orographic right bank is steeper, with stony shores in its lower section and a mix of sand and stony spots in its middle section. Muddy bottoms form the inlet section of the reservoir on both shores. Seasonal water level fluctuation between 2014 and 2016 was marginal, not exceeding 0.5 m and the flooded area ranged between 1.27 and 1.31 km², since the Vltava River Authority aims to maintain high water levels across seasons. In 2015, because of a planned dam reconstruction, the water level decreased by 1.5 m below the normal operating level in late April, lasting over the whole growing season, and the flooded area ranged between 1.24 and 1.25 km² (Fig. 1). In 2016, the reservoir recovered its normal water level^7^.

### Field sampling

The most abundant animal species were sampled each year (from 2014 to 2016) in the first week of August, following the sampling protocol described in Veselý et al.^13^ Among these, the adults of two fish species (the predatory European perch Perca fluviatilis Linnaeus 1758 (*N* = 19) and the omnivorous roach Rutilus rutilus Linnaeus 1758 (*N* = 23)) were collected by angling and gillnetting, while adults of the omnivorous noble crayfish (Astacus astacus Linnaeus 1758 (*N* = 29)) were caught manually with handheld nets and by scuba diving, as well as with baited traps placed along the shoreline in the late afternoon and checked the following morning. Macrozoobenthos were collected using kick netting. The list of macrozoobenthos species (predatory (*N* = 20) and detritivorous macrozoobenthos (*N* = 14)) used for study purposes is in Table 1. All samples were placed on dry ice immediately after collection and then transferred to the laboratory, where they were stored in a freezer (− 30 °C) until further processing for δ¹³C and δ¹⁵N stable isotope analysis (SIA). Subsequently, a sample of white dorsal muscle of fish and of the abdominal muscle of crayfish were used for SIA as recommended by Stenroth et al.^34^ Zoobenthos species were determined and assigned to trophic guilds (i.e., detritivores or predators) based on the literature, and analyzed as whole organisms.

**Table 1:** List of predatory and detritivorous zoobenthos species and zoobenthos species used as baseline for carbon correction caught in studied years.

Trophic guilds	Species	Presence		
		2014	2015	2016
Predatory zoobenthos	*Erpobdella octoculata*	yes	yes	yes
	*Helobdella stagnalis*	yes	yes	yes
	*Lacophilus hyalinus*	no	yes	yes
	*Nebrioporus elegans*	no	yes	no
	*Sympetrum* sp.	no	no	yes
	*Hemiopis sanguisuga*	yes	yes	yes
	*Hygrotus versicolor*	yes	yes	yes
	*Nepa cinerea*	no	yes	no
Detritivorous zoobenthos	*Ceanis* sp.	yes	yes	yes
	*Cloeon dipterum*	yes	yes	yes
	*Ephemera vulgata*	yes	yes	yes
	*Chironomidae*	yes	yes	yes
	*Oligochaeta*	yes	yes	yes
	*Tabanidae* sp.	no	yes	no
Baseline	*Gyralus allsus*	no	yes	yes
	*Lymnea peragra*	no	no	yes
	*Pisidium sp.*	yes	yes	yes
	*Radix ovata*	yes	yes	yes

In addition, during 2014 to 2016, we caught other fish species and another macrozoobenthos functional group. However, these species/trophic guilds were not caught in sufficient numbers in all years. Thus, these species were not included in analyses. In terms of fish, we caught: pike (*Esox lucius*, Linnaeus 1758), asp (*Aspius aspius*, Linnaeus 1758), European catfish (*Silurus glanis*, Linnaeus 1758), burbot (*Lota lota*, Linnaeus 1758), and rudd (*Scardinius erythrophthalmus*, Linnaeus 1758). In addition, we also caught other macrozoobenthos trophic guilds such as filter-feeders, shreders, and collectors. Similarly, we did not catch enough across studied years. Thus, this trophic guilds were excluded from analysis. Only filter-feeders, shreders were used as baseline for further statistical analysis. Their species composition can be found in Table 1.

### Stable isotope analyses

Before SIA, all samples were dehydrated at 50 °C for 48 h and ground to a fine homogenous powder, of which approximately 0.6 mg was weighed into tin capsules with an electronic microbalance. The analyses were performed using a Carlo Erba Flash EA 1112 elemental analyser connected to a Thermo Finnigan DELTAplus Advantage continuous-flow isotope ratio mass spectrometer (Thermo Electron Corporation, Waltham, MA, USA).

Vienna Pee Dee Belemnite and atmospheric N₂ were used as reference standards for δ¹³C and δ¹⁵N, respectively. To control instrument stability, muscle of the Northern pike (Esox lucius Linnaeus 1758) of known isotopic composition was analyzed every six samples. Results are expressed using the conventional δ notation as per mil (‰) difference from the international standards. The analytical precision was < 0.1‰ for δ¹³C and < 0.3‰ for δ¹⁵N.

### Statistical analyses

Prior to the statistical analysis, stable isotope values were standardized using the formulas below. The macrozoobenthos used as baseline over the studied years is in Table 1.

$$\begin{gathered} \delta {\mathrm{13Ccor}}\, = \,\left( {\delta {\mathrm{13Ci}}{-\!\!-}{\mathrm{mean}}\delta {\mathrm{13Ccons}}} \right){\text{ }}/{\text{ range}}\left( {\delta {\mathrm{13Ccons}}} \right); \hfill \\ \delta {\mathrm{15Ncor}}\, = \,\delta {\mathrm{15Ni}}{-\!\!-}\delta {\text{15Nbaseline }}/{\text{ TDF}})\, + \,{\mathrm{2}}, \hfill \\ \end{gathered}$$

where δ13Ci and δ15Ni are the individual values, meanδ13Ccons is the mean δ13C value across consumers in each year (δ13Ccons) is the difference between the maximum and minimum δ13C values across consumers in each year, δ15Nbaseline is the mean δ¹⁵N of filter-feeders and shredders in the studied years, and TDF is set as 3.23.

The isotopic niche area of all consumers was calculated as the Bayesian standard ellipse area (SEAb; encompassing 95% of the data points) and the corrected standard ellipse area (SEAc; considering 40% of central data points) using the R package SIBER^10^. All models converged with values ranging 1.01–1.09 using Gelman and Rubin’s convergence diagnostic. The model values were: Markov Chain Monte Carlo simulation = 1000, number of chains = 3, number of iterations = 20,000, thinning rate = 10.

In general, SEAb provides a measure of the isotopic niche space (i.e., the δ-space) occupied by the sampled population, while SEAc provides a measure of the “core” isotopic niche. To assess the probability of the niche area of each group differing among years, we calculated the proportion of times that the values in the SEAb posterior distribution for one year were lower than for another one^35^. In addition, the ratio of SEAc for each year compared to the first one (i.e., 2014, as a reference) was computed for each group (i.e., species or trophic guild) separately to assess differences among years. All statistical analyses were performed using R version 4.3.3^36^. (https://www.r-project.org/).

## Results

**Fig. 2:**
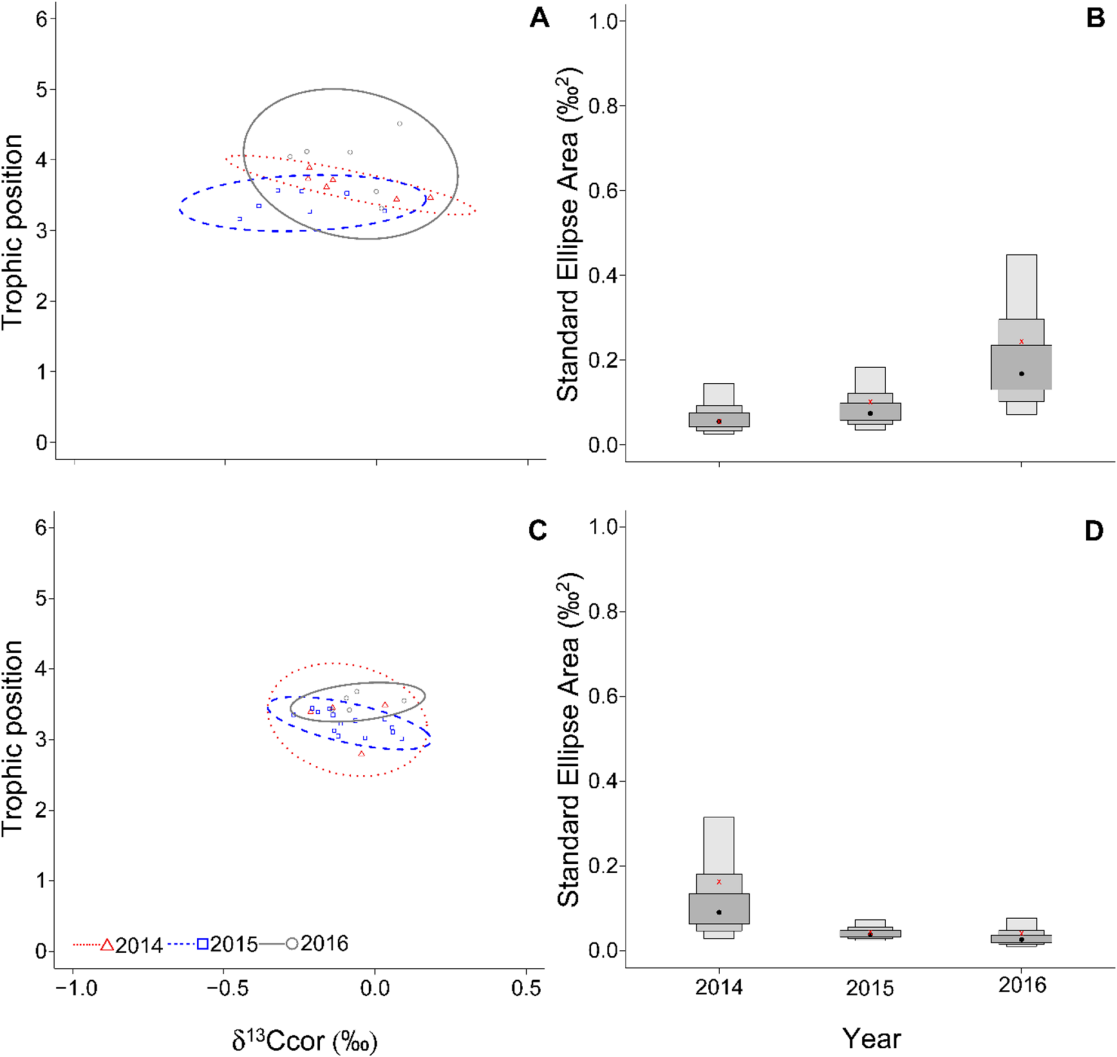
Standard ellipse area (SEAb) representing the trophic niche area of the European perch (**A**) and Roach (**B**), and 95% Bayesian standard ellipse area (SEAb) visualized as boxplots representing the trophic niche area of the European perch (**B**) and roach (**D**) in Nýrsko reservoir over the three years. Dark grey, light grey and outer light grey boxes represent 50%, 75% and 95% confidence intervals, respectively. In the boxplots, the red cross marks the SEAc, while the black dot marks the SEAb mode for each species.

**Fig. 3:**
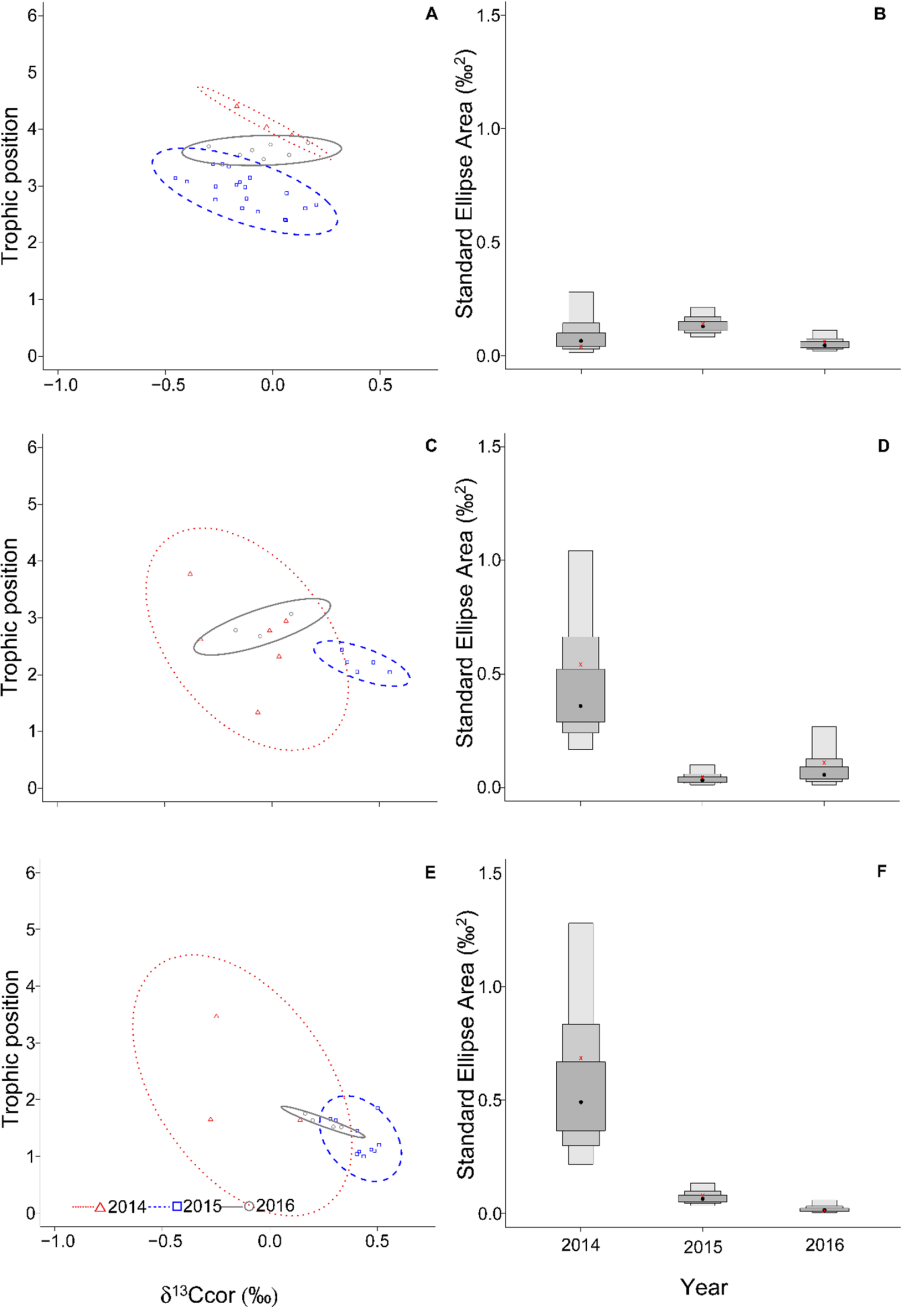
Standard ellipse area (SEAb) representing the trophic niche area of the noble crayfish (**A**), predatory zoobenthos (**C**), and detritivore zoobenthos (**E**). Also, standard ellipse area (SEAb) visualized as boxplots representing the trophic niche area of the noble crayfish (**B**), predatory zoobenthos (**D**), and detritivore zoobenthos (**F**) in Nýrsko reservoir over the three years. Dark grey, light grey and outer light grey boxes represent 50%, 75% and 95% confidence intervals, respectively. In the boxplots, the red cross marks the SEAc, while the black dot marks the SEAb mode for each species.

The effect of water level fluctuation on consumers’ isotopic niche area was group-specific (Figs. 2 and 3). The isotopic niche of perch was narrowest in 2014, and it increased over the following years (Fig. 2A-B), with SEAc being almost twice and five times higher in 2015 and 2016, respectively, compared to 2014 (Table 1). The SEAb posterior distribution of perch in 2016 had 97% probability of being higher than 2014 and 93% probability of being higher than 2015, while the probability of having higher values in 2015 than 2014 was 68% (Table 2). On the contrary, roach had the widest isotopic niche in 2014 and in the following years it shrank (Fig. 2C-D), with SEAc decreasing to 27% in 2015 and 25% in 2016 in comparison to 2014 (Table 2). The roach probability of SEAb posterior distributions in 2015 to be higher than in 2014 was 27%, while the probability of being higher in 2016 than 2014 and 2015 was 4 and 6%, respectively (Table 1).

**Table 2:** Probability of the posterior distribution of bayesian standard ellipse areas (SEAb) of each consumer group for one year to be lower than for another one, and ratios of the bayesian standard ellipse area (SEAb) mode of each year compared to 2014 for each consumer group.

Consumer group	Functional group	SEAc (compared to 2014)			*P* (SEAb 2014 < 2015)	*P* (SEAb 2014 < 2016)	*P* (SEAb 2015 < 2016)
		2014	2015	2016			
*Perca fluviatilis*	predator	1	1,85	4,46	0.68	0.97	0.93
*Rutilus rutilus*	omnivore	1	0,27	0,25	0.03	0.03	0.31
*Astacus astacus*	omnivore	1	3,51	1,55	0.73	0.23	0.03
Predatory zoobenthos	predator	1	0,08	0,20	< 0.001	0.02	0.82
Detritivorous zoobenthos	detritivore	1	0,12	0,02	0	0	0.03

In crayfish, during normal water level regime (2014 and 2016) the isotopic niche was rather narrow compared to 2015, when the water level dropped and the isotopic niche considerably expanded its width (Fig. 3A-B). Particularly, SEAc showed a 3.5-fold increase in 2015 and a three-fold increase in 2016 compared to the first year (Table 2). The SEAb posterior distribution values in 2015 were also always higher than 2014, while the probability of having higher values in 2016 than 2014 and 2015 was 73% and less than 3%, respectively (Table 2). Both predatory and detritivore zoobenthos in 2014 had a wider isotopic niche in comparison to 2015, when their isotopic niche drastically shrank (Fig. 3C-F), with SEAc being approximately one tenth than in 2014 (Table 2). Indeed, the probability of SEAb distributions being higher in 2015 and 2016 than in 2014 was only 0.1-2% in both cases (Table 1). In 2016, the niche area of predatory zoobenthos slightly increased (Fig. 3C-D), with the probability of SEAb values being higher in this year than in 2015 being 82% (Table 1). On the other hand, the niche of detritivore zoobenthos further decreased in 2016 (Fig. 3E-F), with the SEAb values being always higher in 2014 than 2015 and 2016, with a probability of just 0.1% for SEAb values to be higher in 2016 than 2015 and SEAc values in 2016 reaching 3% of SEAc in 2014 (Table 2). To support our results, stable isotopes values of benthic algae and detritus are present in Table 3. While 2014 and 2015 had similar values. Year 2016 is different in both carbon and nitrogen.

**Table 3:** Benthic algae and detritus isotopic values.

	2014		2015		2016	
	δC^13^	δN^15^	δC^13^	δN^15^	δC^13^	δN^15^
Benthic algae	− 12.23+/− 3.07	0.32+/− 1.31	− 14.08+/− 3.25	0.18+/− 0.57	− 26.42+/− 0.77	4.39+/− 2.51
Detritus	− 28.05+/− 1.33	− 0.84+/− 1.67	− 26.87+/− 1.06	0.56+/− 1.69	− 24.84+/− 5.72	0.96+/− 0.98

## Discussion

Abiotic variables play a crucial role for consumers inhabiting an ecosystem, and their fluctuation can be crucial for the whole community^2,37^, determining different outcomes in different species^37^. Water level fluctuation might be such a case. In natural ecosystems, changes in water level during the year (due to floods, droughts, etc.) are predictable for the whole biota inhabiting them, as they are adapted to such cyclic changes^38^. However, extreme environmental anomalies represent an exception^39^. Here, we assessed whether an anthropogenic event that led to strong water level fluctuations affected the isotopic niche area of multiple consumers from different taxa and guilds.

The number of sources used by a consumer defines its isotopic niche area^1,9,17^. Liao et al.^30^ reported that fish from the Three Gorges Dam and Reservoir on the Yangtze River, China, reacted differently to water level fluctuation based on their trophic guild. The authors found that increasing water level increased the isotopic niche area of herbivorous and invertivorous fish species, while it decreased the isotopic niche area in predatory fish. Similar patterns were observed by Qiu et al.^31^ in the Yangtze River oxbow, China, and by Ma et al.^32^ in a Chinese freshwater lake. Azevedo et al.^40^ in an Amazon floodplain lake found that herbivorous fish had a broader isotopic niche with high water levels, while detritivorous and piscivorous fish had a broader isotopic niche during low-water periods. In our study, the isotopic niche area of predatory fish (perch) increased across years, regardless of water level fluctuation. Although in 2015, when the water level dropped, perch followed the same pattern found by Liao et al.,^30^ in the following year (with normal water level) the isotopic niche area was wider than in 2015. We speculate that, while in 2015 this increase might be due to reduced prey availability, leading to increased opportunism, in 2016 this increase could be due to the consumption of juvenile fish after a successful spring spawning together with littoral habitat accessibility.

In addition, Liao et al.^30^ and Qiu et al.^31^ suggested that increasing water level had a slightly negative effect on detritivorous fish and a unimodal effect on omnivorous fish. Our findings contrast with the results of these authors for omnivorous fish, as the isotopic niche area of roach shrank in 2015 and, instead of recovering the following year, it shrank further. These results may be attributed to a reduction in food source availability in 2015^7^, followed by a trophic resilience observed in 2016. This is likely due to the size of the Nýrsko reservoir, as water level fluctuations might have detrimental effects on both habitat accessibility and recovery. This interpretation is partly in line with the results of Costa-Pereira et al.,^41^ who observed a narrower isotopic niche in the dry season, as a result of food source paucity, and a broader isotopic niche in the wet season of a fish species in the Pantanal wetlands. However, omnivorous fish have been shown to change their diet while keeping a similar isotopic niche breadth in response to water level fluctuations^22^.

In a previous study on the same locality, Veselý et al.^7^ found that consumers’ δ¹³C was strongly affected by the water level fluctuation, with the water level drop making the shallow littoral zone inaccessible. This probably made terrestrial detritus unavailable for detritivorous zoobenthos, resulting in a trophic subsidy to benthic algae from deeper zones as an alternative food source. The reduced variation of carbon sources could explain the strong decrease in isotopic niche area observed in both detritivorous and predatory zoobenthos in 2015 and, to a lesser extent, in 2016. This pattern aligns with the one observed in roach in this study, supporting our interpretation.

On the contrary, our results indicated that crayfish showed an opposite trend: a substantial increase in the isotopic niche area in the year when the water level dropped. Such a trend is probably the result of the ecological features of crayfish, which are highly mobile organisms, capable of using a large variety of food sources^42–44^, but also of migrating to deeper zones and using sources from varying depths^13,45,46^. Such abilities probably allowed crayfish to withstand the water level drop by adopting a more generalist feeding strategy, compensating for reduced prey availability using different food sources and habitats. As the water level returned to the original level, the isotopic niche area of crayfish decreased, indicating a shift toward a more specialist diet. However, the isotopic niche in 2016 was still less than a third of that in 2015, confirming that the recovery of trophic behavior was not complete after one year.

Unlike the expansion of the trophic niche observed in crayfish under low water level, less motile organisms such as predatory and detritivorous zoobenthos exhibited a marked reduction in their trophic niche areas. Interestingly, the zoobenthic organisms also showed a shift in their trophic niches toward more enriched carbon values, suggesting changes in diets and habitat use in response to reduced water level. Mao et al.^47^ observed a general trend among invertebrates and fish, where increasing water level led to a shift in carbon isotopic values toward more depleted signatures, suggesting a greater reliance on pelagic-based energy sources under high water conditions. In our study, the drop in water level likely reduced the availability of basal resources, leading to a contraction of the trophic niche areas of predatory and detritivorous zoobenthos and a shift toward enriched carbon values. This pattern suggests a more specialized feeding behavior, likely relying more on algal biofilm, as indicated by the carbon signature of this basal resource (Table 3). Additionally, during the year of low water level, the trophic niches of crayfish, predatory, and detritivorous zoobenthos occupied somewhat lower trophic positions. This suggests a shift in habitat use and diet under these conditions, supporting our hypothesis that these organisms increasingly relied on basal food resources such as algal biofilm.

A previous study^28^ found that most fish species had relatively higher trophic levels in the dry season compared to the wet season, likely due to the increase in fish densities that promoted the presence of piscivorous fish. However, this pattern was not observed in our study, as the two fish species studied maintained similar trophic positions regardless of water level. Our findings are instead consistent with those of Yang et al.,^48^ who reported that the trophic positions of the fish species studied were not significantly affected by fluctuations in water level.

Some limitations of our study come from the temporal and spatial scale, since it was carried out over three years in a single ecosystem. While it is very difficult to study such peculiar conditions in multiple ecosystems at the same time, it would be interesting to sample over a longer term to detect longer-term recovery or delayed effects on trophic structure. Also, the small sample size of some groups means that some results should be taken with caution and interpreted within their context. Finally, although we did not detect any other change across years besides the water level change, and are therefore confident in our results, we cannot completely exclude that other abiotic or biotic factors (e.g., aquatic vegetation, nutrient content, or animal species composition) may have played a role in the observed patterns.

## Conclusion

In conclusion, while water level fluctuation affected each group differently, some similar patterns were observed. Although more investigations are needed, our study provides evidence that water level fluctuations have either positive or negative effect on the isotopic niche area across different taxa and trophic guilds. Moreover, our study suggests that one year is probably not sufficient for an ecosystem such as the one studied here to fully recover its trophic web complexity after an abrupt water level change. Since the frequency of extreme events is increasing under climate change^49,50^, our results are of utmost importance for food web ecologists to proxy their effects at the community level.

## Data Availability

All data used in this manuscript are available in Veselý et al. 2023, Doi: https://doi.org/10.1038/s41598-023-30849-9.
